# Sex-specific predictive ability of SARC-F and SARC-CalF for ultrasound-based sarcopenia in older adults

**DOI:** 10.1186/s12877-026-07510-x

**Published:** 2026-04-18

**Authors:** Ayse Fadiloglu, Eda Ceker, Esra Cataltepe, Fatih Gungor, Nermin Karakurt, Hacer Dogan Varan

**Affiliations:** 1 Department of Geriatric Medicine, Osmaniye Training and Research Hospital, Osmaniye, 80000 Turkey; 2 Department of Geriatric Medicine, Etlik City Hospital, Ankara, Turkey; 3 Department of Geriatric Medicine, Beyhekim Training and Research Hospital, Konya, Turkey; 4 Department of Geriatric Medicine, Gulhane Training and Research Hospital, Ankara, Turkey; 5 Division of Geriatric Medicine, Department of Internal Medicine, Gazi University Faculty of Medicine, Ankara, Turkey

**Keywords:** SARC-F, SARC-CalF, Sarcopenia

## Abstract

**Background:**

The anterior thigh muscles are among the first to be affected in sarcopenia. This study aims to evaluate the sex-specific predictive value of SARC-F and SARC-Calf, in detecting sarcopenia using ultrasound-derived anterior thigh muscle thickness (ATMT) as the diagnostic reference.

**Methods:**

This cross-sectional study included 347 (227 female and 120 male) older adults recruited from a geriatrics outpatient clinic. Sarcopenia was diagnosed based on the presence of low handgrip strength and a low anterior thigh muscle thickness index (ATMT/BMI). The SARC-F and SARC-CalF questionnaires were administered, using both standard (< 31 cm) and population-specific (< 33 cm) calf circumference cut-offs. Anthropometric and ultrasound measurements were performed by trained geriatricians to minimize measurement bias. The screening performance of SARC-F and SARC-CalF was evaluated using receiver operating characteristic (ROC) curve analysis. Multivariate logistic regression models were applied to identify independent predictors of sarcopenia, adjusting for age, sex, BMI, and frailty. Odds ratios (ORs) with 95% confidence intervals (CIs) were reported.

**Results:**

Of the participants, 21.3% were classified as sarcopenic. Sarcopenic individuals exhibited smaller arm circumferences, slower gait speeds, and a higher prevalence of frailty. SARC-F positivity was observed in 76 patients (21.9%), while SARC-CalF positivity was found in 41 patients (11.8%) using the population-specific cut-off (< 33 cm) and in 123 patients (35.4%) using the standard cut-off (< 31 cm). Although SARC-CalF < 33 cm improved sensitivity, the < 31 cm cut-off demonstrated relatively higher specificity, with no significant difference in overall diagnostic performance between the two thresholds. ROC analysis revealed that SARC-F had the highest diagnostic accuracy in the overall population (AUC = 0.693) and women (AUC = 0.708), with good sensitivity and specificity. SARC-CalF < 33 improved sensitivity in both women (72.09% vs. 58.11%) and the overall population (62.16% vs. 58.11%). In men, SARC-CalF < 33 achieved the highest specificity (73.03%), while SARC-F had the highest sensitivity (83.87%). Logistic regression analysis showed that older age, male sex, and frailty were independent predictors of sarcopenia, while SARC-F remained the most predictive screening tool overall. No significant differences in diagnostic superiority were found between tests, though SARC-F had the highest AUC overall.

**Conclusion:**

SARC-F demonstrated the highest diagnostic accuracy and negative predictive value, making it a reliable general screening tool, while population-specific calf circumference adjustments (< 33 cm) enhanced the sensitivity of SARC-CalF, particularly in women.

**Supplementary Information:**

The online version contains supplementary material available at 10.1186/s12877-026-07510-x.

## Introduction

Sarcopenia, a condition characterized by the progressive loss of muscle mass and function, is associated with adverse health outcomes, including frailty, falls, and increased mortality in older adults. Additionally, sarcopenia places a significant burden on healthcare systems by increasing hospitalization rates and prolonging hospital stays [1]. Early diagnosis is crucial for implementing timely interventions, such as resistance exercise, protein supplementation, and vitamin D optimization, to preserve muscle health and mitigate the adverse effects of sarcopenia [2]. Despite the growing emphasis on functional measures such as grip strength, the quantitative assessment of lean mass remains essential for evaluating sarcopenia in geriatric patients. While computed tomography (CT) and magnetic resonance imaging (MRI) are considered the gold standards for muscle mass measurement, their high costs and limited accessibility often make dual-energy X-ray absorptiometry (DXA) and bioelectrical impedance analysis (BIA) more practical alternatives.

However, these methods have limitations in accuracy, particularly in clinical settings where factors such as hydration status and mobility restrictions can affect results [1, 3]. Muscle ultrasound is emerging as a promising, non-invasive, and cost-effective tool for sarcopenia screening, demonstrating a strong correlation with MRI findings [4]. Its ability to assess both morphological and qualitative muscle characteristics, along with its accessibility and ease of use, offers significant clinical advantages. Specifically, anterior thigh muscle thickness (ATMT), assessed by ultrasound and adjusted for body mass index (BMI), has shown promise in detecting sarcopenia. However, further validation studies and standardized operator training are necessary to ensure reliable implementation in clinical practice [4, 5].

A recent study has proposed standardized anatomical landmarks and measuring points for assessing 39 muscles [6]. The anterior thigh, particularly the quadriceps femoris, is a large and easily accessible muscle group that undergoes significant atrophy with aging. Regional variations in age-related muscle loss have been documented, with sarcopenia more frequently diagnosed when defined by reduced anterior thigh muscle thickness compared to other anatomical sites [5, 7]. Given these findings, assessing ATMT via ultrasound may be a valuable approach for the early diagnosis of sarcopenia. Recently, ATMT- calculated as the combined thickness of the rectus femoris and vastus intermedius muscles- was incorporated into the STAR Index. This index, determined by dividing anterior thigh muscle thickness by body mass index (BMI), defines low muscle mass using cut-off values of < 1.0 for women and < 1.4 for men, as established by Kara et al. [8]. The anterior thigh region was specifically selected due to its early involvement in age-related muscle loss, its strong association with mobility and functional decline, and its accessibility for reliable ultrasound assessment in clinical practice.

The SARC-F (Strength, assistance with walking, rising from a chair, climbing stairs, and falls) is a validated screening tool for sarcopenia [9, 10], with strong evidence linking it to sarcopenia-related outcomes such as functional decline, hospitalization, quality of life, and mortality [9, 11]. While SARC-F demonstrates high specificity, its low sensitivity limits its standalone utility, highlighting its role in identifying individuals who require further diagnostic evaluation. To address this limitation, the incorporation of calf circumference (CC) into the SARC-F led to the development of the SARC-CalF, a modified screening tool with enhanced sensitivity and specificity [10, 12].

Although SARC-F and SARC-CalF have been extensively validated against total body muscle mass measurements [12], their efficacy in detecting sarcopenia using ultrasound-based assessments remains uncertain. The present study aims to assess the sex-specific diagnostic accuracy of the SARC-F and SARC-CalF questionnaires in identifying sarcopenia diagnosed via ultrasound-based ATMT/BMI measurements. Specifically, we aimed to compare the performance of these tools between men and women. By comparing these screening tools using an ultrasound-based reference method, this study aims to evaluate the diagnostic performance of SARC-F and SARC-CalF in detecting sarcopenia. The findings may contribute to the integration of ultrasound-based assessments into existing sarcopenia evaluation approaches.

## Methods

### Study population

This prospective cross-sectional study was conducted between April 2023–April 2024 in a geriatrics outpatient clinic. Participants were consecutively recruited during routine clinical visits. Individuals aged ≥ 63 years were eligible for inclusion. Only individuals who were able to understand and comply with verbal instructions were included in the study. Patients were excluded if they had advanced dementia, cerebrovascular disease with sequelae, Parkinson’s disease, rheumatoid arthritis, polyneuropathy, were unable to walk, or were using medications affecting muscle function such as corticosteroids. All data were collected during a single clinic visit. Sarcopenia was diagnosed based on the presence of low handgrip strength and a low anterior thigh muscle thickness index (ATMT/BMI). BMI was recorded for all participants to explore its relationship with sarcopenia. Questionnaires were administered by a trained geriatrician while all anthropometric and ultrasound measurements, including ATMT were performed by a separate geriatrician to minimize measurement bias.

The study protocol was reviewed and approved by the institutional ethics committee (decision number 339) and a written informed consent form was obtained from all participants in accordance with the Declaration of Helsinki.

### Sample size calculation

A priori power analysis was conducted before the initiation of the study to determine the required sample size for evaluating the associations between SARC-F, SARC-CalF, and ultrasound muscle measurements. The required sample size was calculated based on expected effect sizes reported in previous studies [12], using G-Power software version 3.1.9.7 [13]. A total sample size of 347 participants provided sufficient power (85.3%) to detect a correlation coefficient of −0.160 between SARC-F and ATMT at a significance level of 0.05. Additionally, this sample size provided nearly 100% power to detect a correlation coefficient of −0.286 between SARC-F and the STAR index.

### Comprehensive geriatric assessment

The entire cohort underwent a comprehensive geriatric evaluation, which included assessments of functional status using the Katz Basic Activities of Daily Living (ADL) scale [14], and the Lawton-Brody Instrumental Activities of Daily Living (IADL) scale [15]. Nutritional status was evaluated with the Mini Nutritional Assessment–Short Form (MNA-SF) [16], while mood was assessed using the Yesavage Geriatric Depression Scale–Short Form (GDS) [17]. Physical frailty was assessed using the Fried frailty phenotype (FFP) [18, 19]. The total score on the FFP ranges from 0 to 5 points where 0 indicates that the person is robust, 1 or 2 points indicate that the person is pre-frail and ≥ 3 points is considered frail.

### Measurement of muscle mass and anthropometric measures

Anthropometric measurements were performed, including weight, height, arm and calf circumference. Weight was measured using a calibrated scale with participants barefoot and wearing light clothing, and height was measured with a stadiometer. Calf circumference was taken at its widest point while participants stood upright. Arm circumference was measured at the midpoint of the upper arm, with participants instructed to slightly elevate and internally rotate their arm to ensure the biceps were relaxed. A non-elastic tape measure was used for all circumference measurements to ensure consistency and accuracy [18].

### Functional measurements

Handgrip strength was measured using a calibrated digital hand dynamometer (Takei Scientific Instruments Co., Ltd., Niigata, Japan). Participants performed three maximum-effort trials with their dominant hand, and the highest recorded value was considered the handgrip strength (HGS). Low muscle strength was defined using cut-off values of 27 kg for males and 16 kg for females, in accordance with the European Working Group on Sarcopenia in Older People (EWGSOP2) [1].

Physical performance was assessed using the 6-meter walking test. Participants were instructed to walk at their usual pace, and the time taken to complete the distance was recorded using a stopwatch. Walking speed was calculated in meters per second (m/s).

### Ultrasound-based sarcopenia assessment

**Fig. 1:**
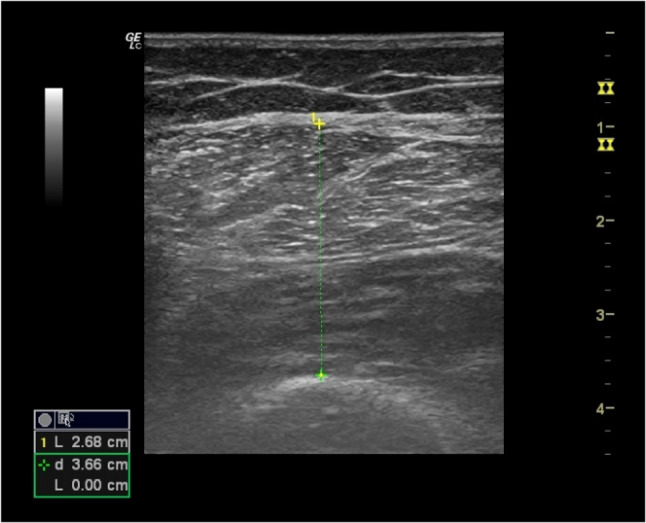
Ultrasound measurement of anterior thigh muscle thickness (ATMT). The measurement represents the combined thickness of the rectus femoris and vastus intermedius muscles at the midpoint between the anterior superior iliac spine and the upper border of the patella

A geriatrician with expertise in musculoskeletal ultrasonography performed the assessments. Anterior thigh muscle thickness (ATMT) was measured using a two-dimensional ultrasonography device (LOGIQ e, GE Healthcare) equipped with a high-frequency linear probe (5–13 MHz). Measurements were conducted with participants in a supine position, focusing on the right thigh. The midpoint between the spina iliaca anterior superior and the upper border of the patella was identified using a tape measure and marked with a pencil for precision. The probe was placed transversely on the marked location, aligned along the long axis of the thigh. Participants were instructed to remain fully relaxed in the supine position throughout the procedure. To avoid soft tissue compression, a generous amount of gel was applied between the probe and the skin, ensuring minimal pressure. Three ultrasound images of the ATMT were captured, and the average value was recorded for analysis (Fig. 1).

The adjustment of ATMT, referred to as the STAR index (ATMT divided by body mass index (BMI)), was calculated to detect low muscle mass, using the established cut-off values of 1.4 for males and 1.0 for females [8]. Patients with both low handgrip strength and low STAR index were classified as sarcopenic.

### Screening sarcopenia with SARC-F and SARC-CalF

The standard SARC-F consists of five items assessing strength, assistance in walking, ability to rise from a chair, ability to climb stairs, and history of falls. Each item is scored from 0 to 2, with higher scores indicating a higher likelihood of sarcopenia. The SARC-CalF includes the same five items as the standard SARC-F, scored similarly, with the addition of a sixth item evaluating calf circumference (CC). The CC item is scored as 0 if the CC is above the established cut-off and 10 if it is below. SARC-F and SARC-CalF were administered using the validated Turkish versions. These instruments are established and previously published sarcopenia screening tools. The Turkish validated versions were used without modification [20].

Participants completed the SARC-F and SARC-CalF questionnaires, with calf circumference cut-off points of < 31 cm (referred to as SARC-CalF-31) and < 33 cm (referred to as SARC-CalF-33). The cut-off is based on prior research that established specific thresholds for identifying low muscle mass and sarcopenia. The < 31 cm cut-off is widely recognized in the literature as an indicator of low muscle mass in both men and women and has been recommended by the EWGSOP and other expert consensus groups. The < 33 cm cut-off has been suggested in some studies as a potentially more sensitive threshold for detecting sarcopenia, particularly in the Turkish population and other specific populations [12, 21]. Comparisons were made between individuals with and without sarcopenia using t-tests and chi-square tests. Given the well-documented physical differences between men and women, sex-specific analyses were performed, emphasizing BMI and its association with sarcopenia.

### Statistical analyses

The statistical analyses were performed using the Statistical Package for the Social Sciences (SPSS) version 26.0 for Windows. The distributions of continuous variables were analyzed using the Kolmogorov–Smirnov test and histograms. The choice of parametric or nonparametric tests was based on the normality of the data distribution. For comparing sarcopenic and non-sarcopenic groups, the Student’s *t*-test was used when the data were normally distributed, whereas the Mann–Whitney *U* test was applied for non-normally distributed data. Similarly, for comparing categorical variables between groups, the Chi-square test was used when the expected frequency in each cell was ≥ 5, while Fisher’s exact test was preferred when the expected frequency was < 5. Spearman’s or Pearson’s correlation coefficients were used to analyze the relationships between two continuous variables. The *p*-value threshold was set to < 0.05 to determine statistical significance in the 95% confidence interval. The correlation analysis between SARC-F/SARC-CalF scores and other parameters has been provided as Supplementary Table 1.

The screening performance (sensitivity, specificity, positive likelihood ratio [+ LR], and negative likelihood ratio [-LR]) of the SARC-F and both SARC-CalF scores, calculated using different calf circumference cut-offs, were evaluated for detecting sarcopenia. Receiver Operating Characteristic (ROC) curve analysis was performed using the MedCalc statistical software v.22.009 to determine the discriminatory power of SARC-F and SARC-CALF in identifying sarcopenic cases based on ultrasound-based muscle mass measurement. The Area Under the Curve (AUC) and 95% confidence interval (CI) were calculated. The comparisons between ROC curves were performed using the DeLong method.

To assess the independent associations between sarcopenia and key demographic and clinical variables, multivariate logistic regression analyses were conducted. Unadjusted and adjusted models were used to examine the relationships between sarcopenia and SARC-F/SARC-CalF scores, along with age, sex, BMI, and frailty status. These variables were selected based on prior literature and their established associations with sarcopenia, as well as their potential role as confounding factors. The results were reported as odds ratios (ORs) with 95% confidence intervals (CIs). In the adjusted models, age, sex, and frailty were included as covariates to determine their influence on sarcopenia diagnosis. The logistic regression results are presented in Table 3.

## Results

This study included 347 participants (227 women, 65.4%), with a mean age of 75.23 ± 6.65 years. Among them, 74 (21.3%) were classified as sarcopenic and 273 (78.7%) as non-sarcopenic. The sarcopenic group had a significantly higher mean age compared to the non-sarcopenic group. There were no significant differences in sex distribution or the prevalence of chronic diseases between the groups. Sarcopenic individuals exhibited significantly smaller arm circumference, lower gait speed, and a higher prevalence of frailty. However, calf circumference did not differ significantly between the sarcopenic and non-sarcopenic groups. General characteristics of the participants, stratified by sarcopenia status, are presented in Table 1.

**Table 1:** General characteristics of the participants according to sarcopenia status

	Total *N* = 347	Non- sarcopenic *N* = 273 (78.7)	Sarcopenic *N* = 74 (21.3)	*p*-value
Age, (years), mean (SD)	75.23 (6.65)	74.47 (6.48)	78.04 (6.52)	< 0.001
Gender, female, n (%)	227 (65.4)	184 (67.4)	43 (58.1)	0.136
Diabetes Mellitus, n (%)	122 (35.2)	90 (33)	32 (43.2)	0.105
Hypertension, n (%)	247 (71.1)	192 (70.3)	54 (73)	0.688
COPD, n (%)	19 (5.5)	15 (5.5)	4 (5.4)	0.617
Coronary Artery Disease, n (%)	83 (23.9)	60 (22)	23 (31.1)	0.107
Height, (cm), mean (SD)	156.57 (9.47)	157.15 (9.36)	154.45 (9.65)	**0.029**
Weight, (kg), mean (SD)	70.47 (13.17)	71.07 (12.89)	68.28 (14.03)	0.107
BMI, (kg/m^2^), mean (SD)	28.84 (5.39)	28.89 (5.33)	28.66 (5.66)	0.740
Arm Circumference, cm, median (IQR)	29 (27–31)	29 (27–31)	27 (25.5–31)	**0.028**
Calf Circumference, cm, median (IQR)	36 (34–39)	36 (34–39)	36 (32.5–39)	0.211
Gait Speed, (m/s), mean (SD)	0.96 (0.30)	1.0 (0.29)	0.8 (0.28)	**< 0.001**
ADL, median (IQR)	6 (5–6)	6 (5–6)	6 (5–6)	0.758
IADL, median (IQR)	8 (7–8)	8 (7–8)	8 (6–8)	**0.012**
MNA-SF, median (IQR)	13 (11–14)	13 (11–14)	12 (10–13)	**0.009**
GDS, median (IQR)	2 (1–5)	2 (1–5)	2 (1–6.5)	0.086
FFP, frail, n (%)	55 (15.9)	31 (11.4)	24 (32.4)	**< 0.001**
SARC-F score, median (IQR)	1 (0–3)	1 (0–3)	3 (1–5)	**< 0.001**
SARC-CalF < 33, median (IQR)	2 (0–4)	1 (0–4)	3 (1–6.5)	**< 0.001**
SARC-CalF < 31, median (IQR)	2 (0–4)	1 (0–3)	3 (1–5)	**< 0.001**

*COPD* Chronic Obstructive Pulmonary Disease, *ADL* Activity of daily living, *IADL* Instrumental activity of daily living, *MNA-SF* Mini nutritional assessment short form, *GDS* Geriatric depression scale, *FFP* Fried Frailty Phenotype

Bold values indicate statistically significant results (*p* < 0.05)

Among the 76 patients with SARC-F positivity, 60 were women and 16 were men. Based on the population-specific SARC-CalF cut-off (< 33 cm), 27 women and 14 men were positive. Using the standard SARC-CalF cut-off (< 31 cm), 92 women and 31 men were identified as positive. Regarding sex differences in the study population, women had a higher BMI and greater arm circumference compared to men; however, calf circumference and sarcopenia did not differ significantly between the sexes. Men exhibited significantly higher HGS and faster gait speed. A comparison of sarcopenia prevalence, anthropometric and muscle strength parameters, and SARC-F/SARC-CalF scores by sex is presented in Table 2.

**Table 2:** Comparison of sarcopenia, anthropometric and muscle strength parameters, and SARC-F/SARC-CalF scores by sex

	Women *N* = 227	Men *N* = 120	*p*-value
Sarcopenia, *n* (%)	43 (18.94)	31 (25.8)	0.136
Height, (cm), Mean (SD)	151.62 (6.5)	165.94 (6.73)	**< 0.001**
Weight, (kg), Mean (SD)	69.08 (13.21)	73.1 (12.74)	**0.007**
BMI, (kg/m^2^), Mean (SD)	30.1 (5.61)	26.47 (4)	**< 0.001**
Arm Circumference, cm, median (IQR)	30 (27–32)	28 (26–30)	**< 0.001**
Calf Circumference, cm, median (IQR)	36 (34–40)	36 (34–38)	0.237
Handgrip Strength, (kg), median (IQR)	16.75 (14.27–19.85)	28 (22.75–32.4)	**< 0.001**
Gait Speed, (m/s), Mean (SD)	0.90 (0.27)	1.07 (0.32)	**< 0.001**
SARC-F, median (IQR)	2 (1–4)	1 (0–2)	**< 0.001**
SARC- CalF < 33, median (IQR)	2 (1–4)	1 (0–3)	**< 0.001**
SARC- CalF < 31, median (IQR)	2 (1–4)	1 (0–2)	**< 0.001**

Bold values indicate statistically significant results (*p* < 0.05)

Logistic regression analysis was performed to examine the association between sarcopenia and key demographic and clinical factors (Table 3).

**Table 3:** Association Between Analyzed Factors and Sarcopenia (Logistic Regression Analysis)

Models	Independent Variables	OR	95% CI	*p*-value
Unadjusted Model	**SARC-F**	**1.38**	**1.22**–**1.57**	**< 0.001**
	**SARC-CalF < 33 cm**	**1.11**	**1.05**–**1.17**	**< 0.001**
	**SARC-CalF < 31 cm**	**1.12**	**1.04**–**1.20**	**0.002**
Adjusted Model 1	**SARC-F**	**1.27**	**1.09**–**1.48**	**0.002**
	**Age**	**1.06**	**1.01**–**1.10**	**0.010**
	**Gender (male)**	**1.96**	**1.05**–**3.64**	**0.033**
	BMI	1.02	0.97–1.08	0.40
	**Frailty Status**	**2.30**	**1.11**–**4.75**	**0.025**
Adjusted Model 2	**SARC-CalF < 33 cm**	**1.07**	**1.00**–**1.15**	**0.040**
	**Age**	**1.06**	**1.02**–**1.11**	**0.003**
	**Gender (male)**	**1.98**	**1.07**–**3.66**	**0.029**
	BMI	1.06	1.00–1.12	0.052
	**Frailty Status**	**2.79**	**1.38**–**5.64**	**0.004**
Adjusted Model 3	**SARC-CalF < 31 cm**	1.06	0.97–1.15	0.22
	**Age**	**1.07**	**1.02**–**1.11**	**0.002**
	**Gender (male)**	**1.94**	**1.05**–**3.59**	**0.035**
	BMI	1.05	0.99–1.11	0.09
	**Frailty Status**	**3.03**	**1.48**–**6.20**	**0.002**

Bold values indicate statistically significant results (*p* < 0.05)

In the unadjusted model, higher SARC-F and SARC-CalF scores were significantly associated with sarcopenia (OR = 1.38, 95% CI: 1.22–1.57, *p* < 0.001 for SARC-F; OR = 1.11, 95% CI: 1.05–1.17, *p* < 0.001 for SARC-CalF < 33 cm; OR = 1.12, 95% CI: 1.04–1.20, *p* = 0.002 for SARC-CalF < 31 cm). In the adjusted models, older age, male sex, and frailty remained significant predictors of sarcopenia across all definitions. Specifically, for SARC-F, the adjusted odds ratio was 1.27 (95% CI: 1.09–1.48, *p* = 0.002), and for SARC-CalF < 33 cm, it was 1.07 (95% CI: 1.003–1.15, *p* = 0.040). Frailty showed the strongest association, with adjusted odds ratios ranging from 2.30 (95% CI: 1.11–4.75, *p* = 0.025) to 3.03 (95% CI: 1.48–6.20, *p* = 0.002) across models. BMI did not show a significant independent association in the adjusted analyses. These results suggest that frailty, age, and sex are key determinants of sarcopenia, while SARC-F remains the most predictive screening tool overall. Additional correlation analysis can be found in Supplementary Table 1. Correlation analysis showed that higher SARC-F and SARC-CalF scores were significantly associated with lower muscle thickness and poorer functional parameters.

The diagnostic performance of the SARC-F and SARC-CalF tools was evaluated across the entire cohort as well as by sex, using population-specific (< 33 cm) and standard (< 31 cm) calf circumference (CC) cut-offs. The ROC analysis for sarcopenia screening tools indicated varying accuracy levels. For the overall population, SARC-F demonstrated the highest area under the curve (AUC = 0.693), with moderate sensitivity (56.76%) and specificity (71.79%). Its negative predictive value (NPV) was 86.0%, indicating its utility in ruling out sarcopenia in the general population. SARC-CalF with the population-specific cut-off (< 33 cm) showed improved sensitivity (62.16%) compared to the standard cut-off (< 31 cm, 58.11%), albeit with slightly lower specificity (64.10% vs. 67.77%).

For the men; SARC-CalF with the population-specific cut-off (< 33 cm) yielded the highest specificity (73.03%) and acceptable sensitivity (61.29%), outperforming other tools in diagnostic accuracy. SARC-F achieved the highest sensitivity (83.87%) in men, highlighting its role in identifying at-risk individuals.

For the women; SARC-F demonstrated the highest diagnostic accuracy (AUC = 0.708) with good sensitivity (67.44%) and specificity (65.22%). The population-specific cut-off (< 33 cm) improved sensitivity (72.09%) in women compared to the standard cut-off (< 31 cm, 58.11%). The results of the ROC analysis are presented in Table 4.

**Table 4:** Receiver Operating Curve (ROC) analysis for SARC-F and SARC-CalF against ultrasound-based sarcopenia

	Cut-off	AUC	SE	*p*	95% CI	Sensitivity	Specificity	+PV	-PV
Overall									
SARC-F	> 2	0.693	0.033	< 0.001	0.642–0.741	56.76	71.79	35.3	86.0
SARC- CalF < 33	> 2	0.674	0.033	< 0.001	0.622–0.723	62.16	64.10	23.4	90.6
SARC-CalF < 31	> 2	0.667	0.033	< 0.001	0.614–0.716	58.11	67.77	24.1	90.2
Men									
SARC-F	≥ 1	0.720	0.051	< 0.001	0.630–0.798	83.87	50.56	37.1	90.0
SARC-CalF < 33	> 1	0.728	0.052	< 0.001	0.640–0.806	61.29	73.03	44.2	84.4
SARC-CalF < 31	> 10	0.679	0.058	< 0.001	0.587–0.761	77.42	51.69	15.1	95.4
Women									
SARC-F	> 2	0.708	0.042	< 0.001	0.644–0.767	67.44	65.22	17.7	94.7
SARC-CalF < 33	> 2	0.653	0.041	< 0.001	0.588–0.715	72.09	56.52	27.9	89.7
SARC-CalF < 31	> 2	0.667	0.033	< 0.001	0.614–0.716	58.11	67.77	17.3	93.9

**Fig. 2:**
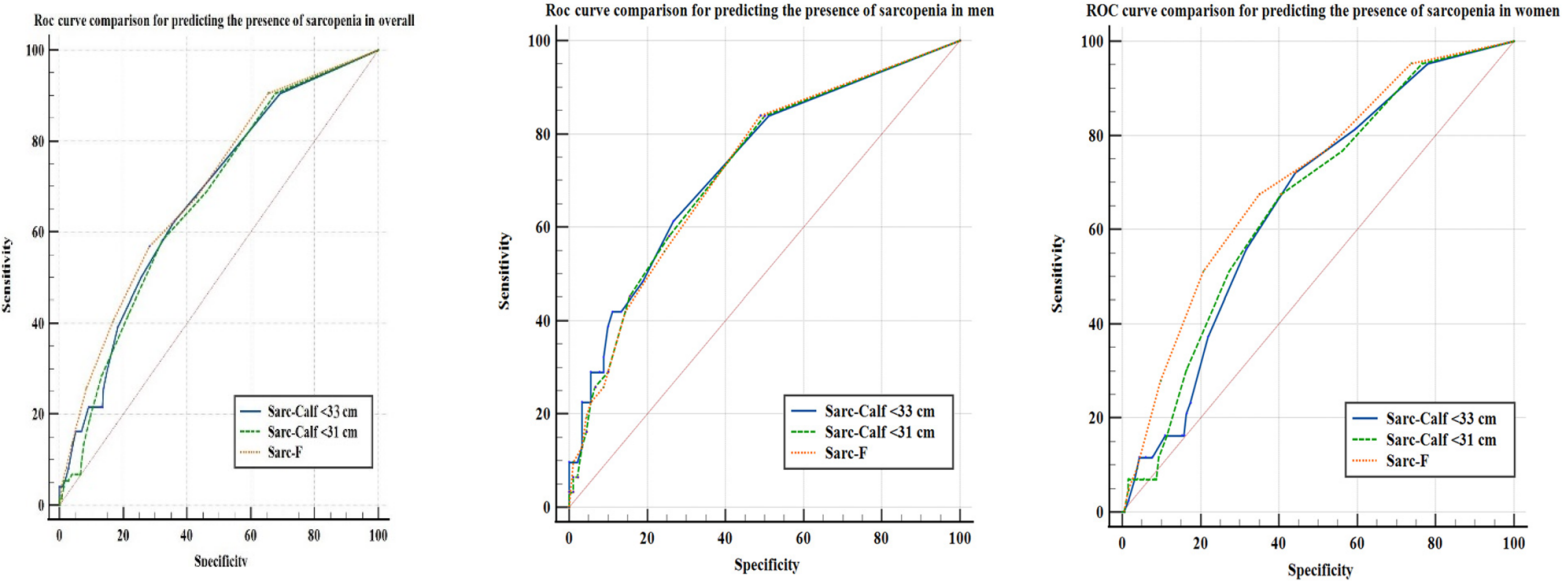
Receiver operating characteristic (ROC) curves of SARC-F, SARC-CalF (< 33 cm), and SARC-CalF (< 31 cm) for the detection of ultrasound-based sarcopenia in the overall population, women, and men

In this study, SARC-F demonstrated the highest AUC value across the total population (0.693) and in women (0.708), suggesting its overall superior diagnostic accuracy. While SARC-CalF < 33 improved sensitivity, particularly in women (72.09% vs. 67.44% with SARC-F), it had lower specificity than SARC-F (56.52% vs. 65.22%). In men, SARC-F showed the highest sensitivity (83.87%), while SARC-CalF < 33 had the highest specificity (73.03%). However, ROC curve comparisons revealed no statistically significant differences between SARC-F and SARC-CalF, indicating that while SARC-F provides the best overall accuracy, SARC-CalF may enhance sensitivity in specific subgroups. (Fig. 2)

## Discussion

This study evaluated the screening performance of the SARC-F and SARC-CalF tools for sarcopenia, using ultrasound-based anterior thigh muscle thickness (ATMT) as the reference criterion. Our findings demonstrate that SARC-F achieved the highest diagnostic accuracy across the general population, with an area under the curve (AUC) of 0.693 and a high negative predictive value (86%). These results align with previous studies validating SARC-F as an effective screening tool for ruling out sarcopenia. Due to its simplicity and moderate sensitivity, SARC-F remains a practical option for initial assessments across diverse populations. In particular, the improved sensitivity and specificity observed in women (AUC = 0.708, sensitivity = 67.44%) suggest that SARC-F may be particularly advantageous in this subgroup, likely due to gender-related differences in muscle mass and fat distribution. Although SARC-F showed numerically higher AUC values, statistical comparisons using the DeLong method did not reveal significant differences between screening tools, suggesting comparable overall diagnostic performance. Therefore, these findings should be interpreted with caution.

The SARC-CalF tool, particularly when using the population-specific calf circumference cut-off (< 33 cm), showed improved sensitivity in both the general population and women compared to the standard cut-off (< 31 cm). This enhancement emphasizes the importance of tailoring cut-off values to population characteristics to optimize diagnostic performance. In men, the population-specific cut-off achieved the highest specificity (73.03%), supporting its utility for confirming sarcopenia in this subgroup. However, SARC-F’s superior sensitivity (83.87%) in men suggests that it remains valuable for identifying at-risk individuals.

Sarcopenia remains a critical public health concern, making early identification through validated, accessible screening tools essential for implementing targeted interventions. A key strength of this study is its use of ultrasound-based ATMT assessment, which provides a non-invasive, accessible, and cost-effective alternative to DXA and BIA for muscle mass evaluation. Unlike whole-body methods, it allows for regional muscle loss assessment, particularly in the anterior thigh, a key site of mobility-related muscle deterioration [23–25]. Prior studies suggest that thigh muscle mass decline can precede whole-body muscle loss, making ATMT a potentially valuable early diagnostic marker [26]. However, ultrasound has certain limitations compared to conventional sarcopenia diagnostic criteria such as EWGSOP2 and AWGS [22]. It has yet to be fully standardized for clinical use, with variations in measurement protocols and the lack of universally accepted cut-off values limiting its comparability across studies. Additionally, ultrasound measurements are operator-dependent, meaning that results can be influenced by the examiner’s expertise and technique. There is also variability in measurement methods across different studies and researchers, which could impact reproducibility and limit widespread adoption in clinical practice. Further research and standardization efforts are needed to fully integrate ultrasound into routine sarcopenia diagnosis.

The ROC curve analysis demonstrated that both SARC-F and SARC-CalF questionnaires exhibited moderate accuracy in identifying sarcopenia when compared to ultrasound-based ATMT/BMI assessments. The AUC value of SARC-F (0.693) falls within the moderate accuracy range, suggesting that while it is useful for initial screening, it should not replace confirmatory diagnostic methods. Clinically, a screening tool should ideally balance sensitivity and specificity to minimize both false negatives and false positives. Our findings indicate that SARC-F has a high negative predictive value (86%), meaning it is effective in ruling out sarcopenia in non-sarcopenic individuals. However, given its moderate sensitivity, it may miss some cases, reinforcing the need for follow-up assessments in at-risk individuals. In clinical settings, SARC-F may be particularly useful for quick, low-resource screenings in primary care or outpatient geriatrics, where a high non-predictive value is valuable for identifying low-risk individuals.

We observed that using a 33 cm cut-off for SARC-CalF increased sensitivity but reduced specificity. This suggests that a higher threshold captures more at-risk individuals by including those with borderline muscle mass but at the expense of misclassifying some non-sarcopenic individuals. The 31 cm cut-off, in contrast, maintains higher specificity, making it more reliable for confirming sarcopenia. Clinically, the 33 cm cut-off may be preferable in settings prioritizing early detection, such as community screenings or preventive health programs, whereas the 31 cm cut-off might be more useful in specialized geriatrics clinics where confirmatory testing is readily available.

A comparable study conducted in Turkey assessed SARC-F and SARC-CalF tools for sarcopenia screening using EWGSOP criteria, with both population-specific (SARC-CalF < 33) and standard calf circumference cut-offs [12]. The study showed that adding the CC item to SARC-F (i.e., SARC-CalF) enhanced specificity and diagnostic accuracy but did not improve sensitivity for identifying sarcopenia among community-dwelling Turkish older adults. In contrast, in our study, the addition of calf circumference (CC) to the original SARC-F tool to create SARC-CalF improved sensitivity in the overall population, with the population-specific cut-off of < 33 cm yielding a slight increase. However, the highest sensitivity in men was achieved with SARC-F, while the highest specificity was observed with SARC-CalF at the population-specific cut-off (SARC-CalF < 33). In women, sensitivity increased only with the population-specific cut-off (SARC-CalF < 33). This finding underscores the importance of tailoring screening tools to local populations, as variations in body composition and demographics significantly impact diagnostic accuracy.

These findings indicate that while SARC-F remains a robust, broadly applicable screening tool, using gender-specific cut-offs or supplementing SARC-CalF measures could improve screening outcomes for specific groups. The use of these localized cut-offs can enhance the sensitivity and specificity of sarcopenia screening, ultimately improving the identification and management of at-risk individuals. This tailored approach may optimize sarcopenia screening in diverse clinical settings, particularly for older adults who are at risk and require early detection and intervention.

Limitations of the study include its cross-sectional design, which precludes longitudinal assessment of muscle decline and sarcopenia progression. Additionally, as our cohort was limited to community-dwelling older adults, the findings may not be generalizable to institutionalized populations or those with significant comorbidities that affect muscle health. Furthermore, ultrasound-based sarcopenia diagnosis is not yet standardized, and measurement techniques may vary across studies and researchers, affecting reproducibility.

One important limitation of the present study is that STAR Index cut-off values were originally derived from a younger population, which may limit their direct applicability to older adults [8]. Nevertheless, accumulating evidence, including our own studies, supports the clinical relevance of anterior thigh muscle thickness (ATMT) and related indices in older adults, with consistent associations with frailty, functional impairment, and sarcopenia [27–30]. Furthermore, recent studies have demonstrated that both anthropometric and machine learning–based approaches incorporating muscle-related parameters enhance geriatric risk stratification [31], and that these measures are also linked to swallowing dysfunction in older populations [32, 33]. However, these findings primarily reflect clinical applicability rather than formal age-specific validation of cut-off values. Accordingly, STAR index thresholds should be interpreted with caution, and future studies are warranted to establish and validate age-specific cut-offs in geriatric populations.

We used the validated Turkish versions of SARC-F and SARC-CalF; however, questionnaire-based assessments may still be subject to reporting bias. Another limitation of this study is that not all potential confounding factors, such as weight and comorbidities including diabetes mellitus, were included in the regression models. Additionally, sex-specific power analyses were not conducted, and the relatively smaller sample size of male participants may have limited the statistical power of subgroup analyses. These variables could influence the relationship between muscle thickness, sarcopenia, and questionnaire-based assessments. While we conducted group comparisons and correlation analyses, regression modeling could have provided a more precise estimation of independent associations by controlling for multiple covariates simultaneously. Additionally, the study did not include a direct comparison with established sarcopenia diagnostic criteria such as EWGSOP2 and AWGS, which limits the ability to assess how ultrasound-based assessments align with these widely used frameworks. Future research should incorporate such comparisons to enhance the clinical relevance and applicability of ultrasound for sarcopenia screening. Despite these limitations, the study contributes to the growing evidence supporting ultrasound as a viable tool for sarcopenia assessment and highlights the need for further research to establish standardized protocols.

From a clinical perspective, SARC-F may be preferred as an initial screening tool in routine practice due to its simplicity and high negative predictive value, while SARC-CalF with a < 33 cm cut-off may be particularly useful in settings where higher sensitivity is desired, especially in women.

## Conclusions

SARC-F demonstrated the highest overall diagnostic accuracy for sarcopenia screening, whereas SARC-CalF using a population-specific < 33 cm cut-off improved sensitivity, particularly among women. Our findings reveal clinically relevant sex-specific differences in the performance of these screening tools, highlighting that sensitivity and specificity vary according to gender. These results support the complementary use of SARC-F and SARC-CalF in clinical practice and emphasize the importance of incorporating sex-specific considerations when selecting screening strategies for sarcopenia.

## Supplementary Information

Supplementary Material 1.

## Data Availability

The datasets generated and/or analyzed during the current study are available from the corresponding author on reasonable request.
