# Supplementary material for: Sex-specific predictive ability of SARC-F and SARC-CalF for ultrasound-based sarcopenia in older adults
Source: BMC Geriatr. 2026 Apr 18;26:773. doi: 10.1186/s12877-026-07510-x (PMC13224712; doi:10.1186/s12877-026-07510-x)
Supplement: Supplementary file 1 — Supplementary Material 1. [file 12877_2026_7510_MOESM1_ESM.docx]

**Supplementary Table 1:** Correlation Analysis Between age, anthropometric and muscle strength parameters, comprehensive geriatric evaluation parameters with SARC-F/SARC-CalF Scores

|  | **SARC-F**  **rho(p)** | **SARC-CalF <31**  **rho(p)** | **SARC-Calf <33 rho(p)** |
| --- | --- | --- | --- |
| Age | 0.254(<0.001) | 0.273(<0.001) | 0.282(<0.001) |
| Weight | -0.072(0.180) | -0.285(<0.001) | -0.192(<0.001) |
| Height | -0.335(<0.001) | -0.337(<0.001) | -0.370(<0.001) |
| BMI | -0.149(0.005) | -0.068(0.207) | 0.038(0.477) |
| Waist Circumference | 0.161(0.003) | -0.025(0.642) | 0.059(0.276) |
| Arm Circumference | 0.027(0.613) | -0.181(0.001) | -0.062(0.249) |
| Handgrip Strength | -0.467(<0.001) | -0.491(<0.001) | -0.487(<0.001) |
| Gait Speed | -0.516(<0.001) | -0.455(<0.001) | -0.500(<0.001) |
| ADL | -0.281(<0.001) | -0.207(<0.001) | 0.238(<0.001) |
| IADL | -0.378(<0.001) | -0.351(<0.001) | -0.351(<0.001) |
| MNA-SF | -0.245(<0.001) | -0.289(<0.001) | -0.287(<0.001) |
| MMSE | -0.220(<0.001) | -0.226(<0.001) | -0.218(<0.001) |
| GDS | 0.313(<0.001) | 0.304(<0.001) | 0.320(<0.001) |
| FFP | 0.463(<0.001) | 0.497(<0.001) | 0.506(<0.001) |
| Clinical frailty scale | 0.495(<0.001) | 0.437(<0.001) | 0.472(<0.001) |
| ATMT | -0.160(0.003) | -0.280(<0.001) | -0.215(<0.001) |

Rho: Spearman's Correlation Coefficient
